# Innovative rapid pathological assessment technology for hepatocellular carcinoma following comprehensive treatment guided by indocyanine green fluorescence imaging

**DOI:** 10.1007/s13304-025-02301-2

**Published:** 2025-07-07

**Authors:** Changcheng Tao, Huiru Yang, Nan Hu, Bo Zheng, Zhiyu Lu, Weiqi Rong, Fan Wu, Liming Wang, Dayong Cao, Jianxiong Wu, Zhanhan Tu, Xuan Meng, Haizhen Lu, Hongguang Wang

**Affiliations:** 1 Department of Hepatobiliary Surgery, National Cancer Center/National Clinical Research Center for Cancer/Cancer Hospital, Chinese Academy of Medical Sciences and Peking Union Medical College, Beijing, 100021 China; 2 Department of Pathology, National Cancer Center/National Clinical Research Center for Cancer/Cancer Hospital, Chinese Academy of Medical Sciences and Peking Union Medical College, Beijing, 100021 China; 3 Robert Kilpatrick Clinical Sciences Building, Leicester Royal Infirmary, University of Leicester, Leicester, UK

**Keywords:** Hepatocellular carcinoma, Comprehensive treatment, Pathological evaluation, Indocyanine green

## Abstract

Background: Traditional pathological techniques for diagnosing liver cancer are limited by the need for examination of multiple points. Preoperative treatment can complicate the evaluation by causing tumor necrosis. The evolving treatment landscape requires precise, integrated, and comprehensive management to address the limitations, specifically in localization, sampling, and speed. This study aims to innovatively explore the use of indocyanine green (ICG) fluorescence imaging to guide accurate and rapid localization of residual cancer foci, facilitating rapid pathological sampling and evaluation, termed as rapid pathological technology. Patients and Methods: In this prospective and observational study, patients undergoing surgical resection for HCC were enrolled. Traditional evaluation involved specimen fixation, sampling, sectioning, and microscopic observation, while rapid evaluation entailed fluorescence-guided rapid pathological sampling and evaluation post-dissection. Results: A total of 37 HCC patients were included, and 103 samples of varying fluorescence intensity were collected. Analysis revealed a significant negative correlation between fluorescence intensity and tumor necrosis ratio (ρ = −0.89, p < 0.001). Furthermore, logistic regression modeling yielded an AUC of 0.947, with a specificity of 0.900 and sensitivity of 0.930, indicating strong discriminatory power for tumor necrosis status. An optimal cut-off fluorescence intensity of ≥ 20.5 was identified for predicting partial tumor necrosis. The Youden index is 0.830. Decision curve analysis demonstrated the clinical utility of the prediction model for guiding treatment decisions regarding tumor necrosis. Conclusions: Rapid pathological evaluation using ICG fluorescence-guided resection specimens in liver cancer is feasible, enabling effective localization of residual cancer foci and enhancing the accuracy and efficiency of pathological sampling.

Trial registration: ChiCTR2400082814

**Supplementary Information:**

The online version contains supplementary material available at https://doi.org/10.1007/s13304-025-02301-2.

## Introduction

Liver cancer is a prevalent malignant tumor that poses a significant threat to human survival. It encompasses three primary pathological types: hepatocellular carcinoma (HCC), intrahepatic cholangiocarcinoma (ICC), and combined hepatocellular-cholangiocarcinoma (cHCC-CC) [1, 2]. These types exhibit significant differences in pathogenesis, biological behavior, histopathology, treatment methods, and prognosis. HCC accounts for 75% to 85% of cases [3]. HCC typically arises in patients with chronic liver disease, particularly in those with cirrhosis induced by alcohol consumption or chronic hepatitis B virus (HBV)/hepatitis C virus (HCV) infection [4]. It often displays varied biological behaviors across different liver diseases. Hepatic resection stands as a potentially curative therapy and is the preferred choice for patients with surgical indications. However, late-stage diagnosis is common because early-stage patients are usually asymptomatic, resulting in a dismal five-year survival rate of 18% [5]. In recent years, numerous international clinical studies, including those on neoadjuvant therapy and conversion therapy [6–8], aim to reduce tumor staging to meet resection standards, thereby improving patient prognosis.

Traditionally, pathological diagnosis of liver cancer involves examining at least seven points within and around the tumor [9]. However, the emergence of various preoperative treatments has rendered this method inadequate, increasing the complexity of pathological diagnosis. Preoperative treatments induce tumor necrosis of varying degrees, necessitating additional pathological sections to accurately assess pathological remission and treatment response, thereby escalating pathologists’ workload and technical requirements. Moreover, identifying and sampling normal and necrotic tumors through visual inspection demands substantial clinical experience.

To address this issue, we have innovatively applied indocyanine green (ICG) fluorescence staining to facilitate rapid pathological sampling. ICG, an organic dye exclusively cleared by the liver, is commonly used clinically to assess liver damage and reserve function [10]. It can be excited by near-infrared light (wavelength 750–810 nm), emitting fluorescence (wavelength ~ 840 nm), with a tissue penetration depth ranging from 0.5 to 1 cm [11], fluorescing upon uptake by hepatocytes. By conducting rapid sampling based on different fluorescence intensities, we aim to alleviate the postoperative pathological assessment challenges faced by patients undergoing neoadjuvant or conversion therapy.

Building upon this innovative approach, the integration of ICG fluorescence staining offers a multifaceted solution to the complex challenges encountered in the in diagnosing and treating liver cancer. ICG, through its selective accumulation in liver tissue, serves as an ideal intraoperative visualization agent, aiding surgeons in identifying tumor margins and preserving healthy liver parenchyma. Furthermore, the application of ICG fluorescence extends beyond surgical boundaries, potentially revolutionizing pathological sampling and evaluation. Traditional pathological assessment methods heavily rely on visual inspection, which may overlook subtle changes in tumor morphology induced by preoperative treatment. By leveraging the unique fluorescent properties of ICG, pathologists can accurately delineate viable tumor areas from necrotic or fibrotic tissues, facilitating precise evaluation of treatment response and residual disease burden. This enhanced diagnostic accuracy not only streamlines postoperative management decisions but also serves as a valuable prognostic indicator for patient survival and disease recurrence [11].

## Materials and methods

### Study design and participants

This prospective and observational study was conducted to recruit patients undergoing surgical resection for hepatocellular carcinoma (HCC) between January 2022 and June 2024 at our hospital. Eligibility criteria included: (1) Age ≥ 18 years, regardless of gender, with voluntary participation; (2) Confirmed diagnosis of hepatocellular carcinoma; (3) Classified as BCLC A-C or CNLC Ia-IIIb before treatment [12, 13]; (4) Underwent neoadjuvant therapy or conversion therapy before surgery. Exclusion criteria comprised: (1) Missing critical clinical data; (2) Infeasibility of surgical treatment; (3) No preoperative ICG test. This study was registered on the Chinese Clinical Trial Registry. Ethical approval was obtained from the Ethics Committee, and all participants provided informed consent following the principles of the Declaration of Helsinki.

Enrollment and assessment of all subjects, including adherence to inclusion and exclusion criteria, was conducted by a multidisciplinary team (MDT) comprising a surgeon, physician, radiologist, pathologist, and other relevant professionals. Subsequently, the MDT collaboratively determined the most appropriate treatment plan for each patient.

### Treatment process

#### Neoadjuvant or conversion therapy

**Fig. 1:**
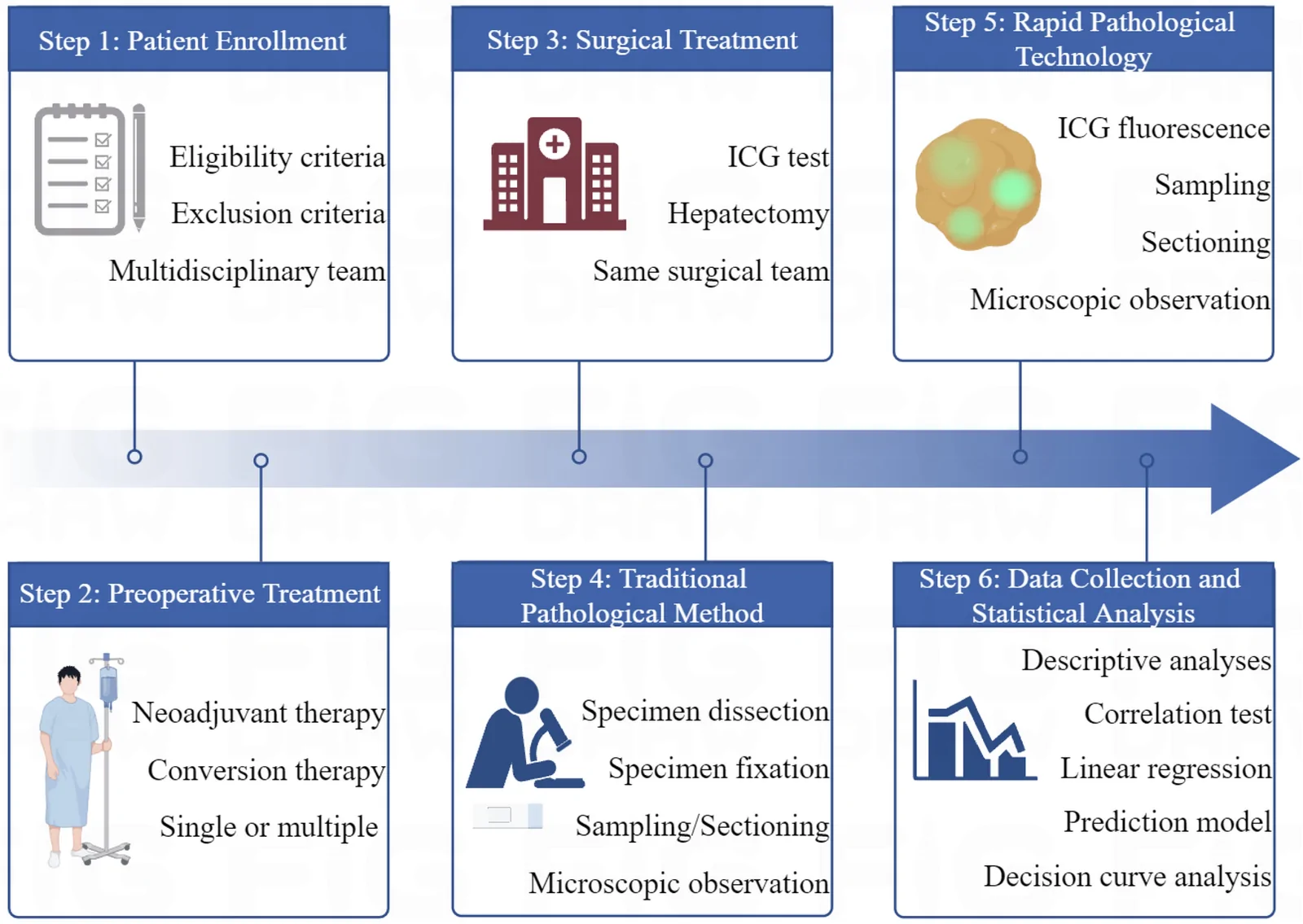
Research flowchart. Drawing by Figdraw

Prior to surgery, all HCC patients underwent either single or multiple neoadjuvant or conversion treatments, including radiotherapy, molecular targeted therapy, transarterial chemoembolization (TACE) and combined therapy. The objectives of preoperative treatment were twofold: first, to serve as conversion therapy, reducing surgical staging, and second, as adjuvant therapy, reducing the high-risk recurrence rate post-surgery. This comprehensive treatment process was clearly defined for the patients. The treatment flowchart is illustrated in Fig. 1.

#### ICG test

One week prior to surgery, all patients underwent a liver reserve function test—an essential assessment for evaluating both the current state of liver function and its potential compensatory capacity. Due to the liver’s remarkable ability to regenerate and compensate, conventional liver function tests may not sensitively detect early-stage functional impairment, as normal physiological function can often be maintained despite underlying damage. In contrast, liver function reserve testing provides a more accurate evaluation of hepatic performance under stress. Clinically, the ICG excretion test is commonly used for this purpose. The recommended ICG dose is 0.5 mg/kg administered via intravenous injection, after which the retention rate of ICG in the bloodstream over a specific time period is measured. This reflects the liver’s capacity to uptake and excrete ICG, thereby indicating its functional reserve. The most commonly used indicator is the 15-min retention rate (ICG-R15), with values less than 10% considered normal and 10%−20% indicating a mild decline in liver reserve function. All HCC patients included in this study had ICG-R15 values below 20%, meeting the inclusion criteria [14, 15].

After intravenous injection, ICG is rapidly taken up by hepatocytes, causing the liver tissue to emit fluorescence. It is then almost entirely excreted through the biliary tract within a few hours [16]. Since ICG does not undergo enterohepatic circulation, its fluorescence intensity in the liver gradually diminishes over time until it disappears [17]. In the preoperative evaluation of patients with HCC, ICG is commonly used as a standard reagent to assess liver function reserve, with liver function status evaluated by monitoring ICG metabolism in the bloodstream. ICG possesses inherent fluorescent properties and can be selectively taken up by normal hepatocytes, exhibiting green fluorescence under near-infrared imaging equipment, thereby enabling real-time visual localization. However, due to abnormal biliary structures in liver cancer tissue, ICG cannot be effectively excreted from tumor areas, resulting in persistent fluorescence within the tumor [15]. This characteristic allows ICG to serve as a tumor marker during liver surgery, aiding in the detection of small lesions that are not visible to the naked eye. Importantly, viable tumor tissue can absorb ICG and exhibit fluorescence, whereas necrotic tissue, having lost its functional capacity, cannot take up ICG and therefore shows no fluorescence signal. Based on this mechanism, ICG fluorescence imaging can be used intraoperatively to rapidly localize residual active tumor tissue and facilitate more accurate pathological assessment. Only patients with normal ICG test results were deemed eligible to undergo surgery. This preoperative evaluation also ensured sufficient ICG uptake by both liver tissue and tumors, enabling effective intraoperative fluorescence imaging [18].

#### Surgical treatment

Prior to surgery, all patients underwent a multidisciplinary team (MDT) discussion, and surgery was typically scheduled 4 to 6 weeks after neoadjuvant or conversion treatments. To maintain consistent operative quality and safety, all procedures were performed by the same surgical team.

Before receiving surgical treatment, patients need to complete imaging examinations (CT and MRI) to evaluate the current state of the tumor (size and necrosis) and the relationship between the tumor and blood vessels. According to the imaging evaluation results, the best and appropriate surgical method was selected, such as anatomical hepatectomy (segmental hepatectomy or combined segmental hepatectomy) or non anatomical hepatectomy.

The extent of hepatectomy was determined by comprehensive evaluation of tumor characteristics and degree of cirrhosis. Surgical approach (open or laparoscopic) was selected based on tumor location and size. At the initial stage of operation, abdominal exploration was performed to exclude distant metastasis. If necessary, intraoperative ultrasound was combined to evaluate the tumor. Intraoperative ultrasonography was required for the quantitative assessment of the extent of the tumor and its relationship with major vascular structures. During the operation, the selective and dynamic region-specific vascular occlusion technique in the liver region can block the left and right hepatic blood flow respectively to control liver bleeding and protect liver function [19]. Surgical methods include anatomic and non anatomic hepatectomy, and the surgical margin was routinely taken after the specimen was isolated and sent to pathological examination to ensure that the surgical margin was negative. In cases of tumor adherence to major vascular occlusion, surgeons carefully dissected and peeled the lesions away from the vascular surface (null-margin resection), with a Cavitron Ultrasonic Surgical Aspirator to avoid cutting the major vessels and prevent postoperative liver failure. After the operation, the abdominal drainage tube was routinely indwelling, and the changes of vital signs were closely monitored.

#### Pathological diagnosis

##### Traditional pathological method

**Fig. 2:**
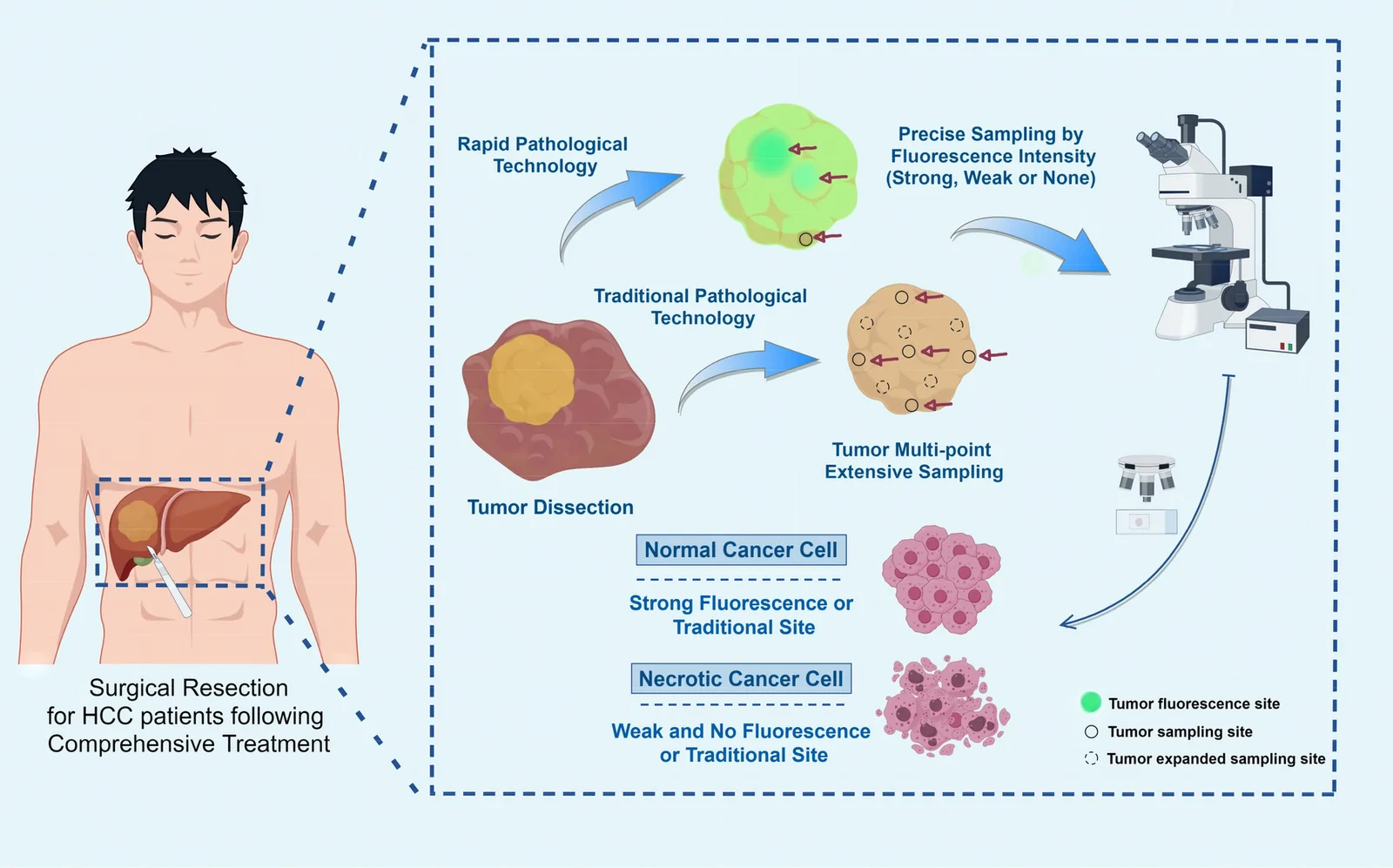
Schematic diagram of traditional and rapid pathological sampling

Upon receipt of the surgical specimen in the pathology department, dissection was performed along the largest surface. Multiple sections were cut parallel to the surface at 1 cm intervals to fully expose the tumor, which was then fixed in a 10% neutral formalin solution (fixation time > 24 h). Liver tumors and surrounding areas indicative of tumor biological behavior were sampled using 7-point sampling method (Fig. 2). Samples were taken 1:1 at the junction of cancer and adjacent liver tissue at the clock positions of 12 o'clock, 3 o'clock, 6 o'clock, and 9 o'clock in the tumor; at least one sample was taken inside the tumor; one piece of liver tissue was taken within a range of ≤ 1 cm (near the cancer) and > 1 cm (far from the cancer) from the edge of the tumor. For small liver cancers with a maximum diameter of ≤ 3 cm for a single tumor, all samples should be taken for examination. The actual location and quantity of materials taken must also be considered based on the diameter and quantity of the tumor [20, 21].

Observation and description of gross specimens focused on various parameters, including size, number, color, texture, relationship with blood vessels and bile ducts, capsule status, surrounding liver tissue lesions, type of cirrhosis, distance from the tumor to the resection margin, and resection margin status. Microscopic observation involved evaluating degree of differentiation, histological morphology, subtype, and growth pattern of liver cancer. The main assessment was the proportion of tumor bed components in the resection specimen: necrotic tumor, surviving tumor, and tumor interstitial (fibrous tissue and inflammation). The total percentage of residual tumor was determined based on the evaluation of these components in each section.

##### Rapid pathological sampling method

Following tumor removal, the tumor was dissected along the maximum diameter by the surgeon. We will assign this subjects to experimental groups. The surgeon did not know that the sample would undergo rapid pathological examination and remained blinded. Pathological sampling was then conducted under the guidance of ICG fluorescence imaging. Sampling observations were based on the fluorescence intensity within the tumor, measured using a fluorescence value instrument (Brand: OptoMedic, averaging five measurements). Sampling was performed at different fluorescence intensities, primarily focusing on tumor type and necrosis rate. Strong fluorescence was defined as > 100 and ≤ 278, weak fluorescence as > 0 and ≤ 100, and no fluorescence as 0. Each case underwent at least one fluorescence-guided sampling (Fig. 2). The samples were processed into pathological sections for microscopic observation, assessing malignant components, necrosis rate, tissue subtypes, and degree of differentiation.

### Sample size

A sample size of 100 tumors provides a two-sided 95% confidence interval with a width equal to 0.170 when the complete tumor necrosis ratio is 25% [22]. Because this was a pilot study, a formal power calculation was not required.

### Statistical analysis

Descriptive analyses were used to characterize the demographic and clinical features of participants. Categorical variables were summarized using frequency and percentage. For continuous variables that followed a normal distribution, the mean and standard deviation were utilized, while those not following a normal distribution were described using the median and interquartile ranges. Postoperative physical complications during hospitalization were evaluated using the Clavien-Dindo Classification [23, 24]. A scatter plot was employed to visualize and initially explore the association between ICG intensity and the tumor necrosis ratio. The Spearman correlation test was conducted to examine the specific correlation between them. Linear regression was employed to investigate the impact of ICG intensity on tumor necrosis ratio. An exploratory prediction model using logistic regression was developed to assess the ability of ICG intensity to differentiate between complete tumor necrosis and partial tumor necrosis. Discrimination of the prediction model was evaluated using the receiver operating characteristic (ROC) curve and the area under the curve (AUC). The calibration of the prediction model was assessed using the Hosmer and Lemeshow (HL) goodness-of-fit test and calibration curve. Decision curve analysis (DCA) was used to evaluate the clinical utility of the prediction model. The significance level for this study was set at α = 0.05.

## Results

### Demographic and clinical characteristics

A total of 37 patients met the eligibility criteria and were enrolled in this study at baseline. Among them, 33 (89.19%) were male; 12 (32.43%) were aged 60 years or older; and 15 (40.54%) were overweight. Cirrhosis was present in 30 (81.08%) patients, with 35 (94.59%) affected by hepatitis B virus (HBV) and 3 (8.10%) by hepatitis C virus (HCV). Regarding treatment, 8 patients received radiotherapy and targeted drug therapy, 2 underwent radiotherapy, targeted drug therapy and TACE. Additionally, 5 patients received radiotherapy and TACE, 22 underwent radiotherapy. Preoperative laboratory examinations revealed ALT levels of 32.30 (22.00, 43.00) U/L, AST levels of 34.50 (28.90, 54.30) U/L, ALB levels of (39.44 ± 4.08) g/L, TBIL levels of 15.40 (12.70, 20.60) μmol/L, and AFP levels of 10.54 (5.00, 252.00) ng/mL, as shown in Table 1.

**Table 1:** Baseline demographics and clinical characteristics in patients

Characteristic	Patients (n = 37)
Sex (Male)	33 (89.19%)
Age (≥ 60)	12 (32.43%)
Overweight (> 24 kg/m^2^)	15 (40.54%)
HBV	35 (94.59%)
HCV	3 (8.10%)
Cirrhosis	30 (81.08%)
Preoperative treatment	
Radiotherapy and Targeted drug therapy	8 (21.62%)
Radiotherapy, Targeted drug therapy and TACE	2 (5.41%)
Radiotherapy and TACE	5 (13.51%)
Radiotherapy	22 (59.46%)
Recist	
PR	8 (21.62%)
CR	1 (2.70%)
SD	28 (75.68%)
mRECIST	
PR	18 (48.65%)
CR	3 (8.10%)
SD	16 (43.24%)
Preoperative examination	
ALT (U/L)	32.30 (22.00, 43.00)
AST (U/L)	34.50 (28.90, 54.30)
ALB (g/L)	39.44 ± 4.08
TBIL (μmol/L)	15.40 (12.70, 20.60)
AFP (ng/mL)	10.54 (5.00, 252.00)

*HBV* Hepatitis B virus, *HCV* Hepatitis C virus, *TACE* Transcatheter arterial chemoembolization, *PR* Partial Response, *CR* Complete response, *SD* Stable disease, *AFP* α-fetoprotein

### Surgery and complications

All 37 patients underwent successful surgery with a mean surgical duration of 251 (175, 356) minutes and intraoperative bleeding of 350 (100, 650) mL. Postoperative complications, ranging from Clavien-Dindo grade I to grade II, occurred in 36 patients, while 1 patient experienced a grade IV complication. All 37 patients recovered smoothly and were discharged without complications.

### Traditional pathological method

Among the 37 patients diagnosed with HCC, microvascular invasion (MVI) was observed in 19 patients. Disease staging revealed 16 patients at BCLC stage A, 9 at BCLC stage B, and 12 at BCLC stage C. Furthermore, 1 patient was classified as CNLC stage Ia, 14 as CNLC stage Ib, 4 as CNLC stage IIa, 10 as CNLC stage IIb, 6 as CNLC stage IIIa, and 2 as CNLC stage IIIb. Additionally, 32 patients had a single tumor, while 5 had multiple tumors. Liver capsule invasion was present in 24 patients, with a maximum tumor diameter of 6.00 (4.00, 9.80) cm. Complete pathological response (CPR) was observed in 3 patients following comprehensive sampling.

### Rapid pathological sampling method

**Fig. 3:**
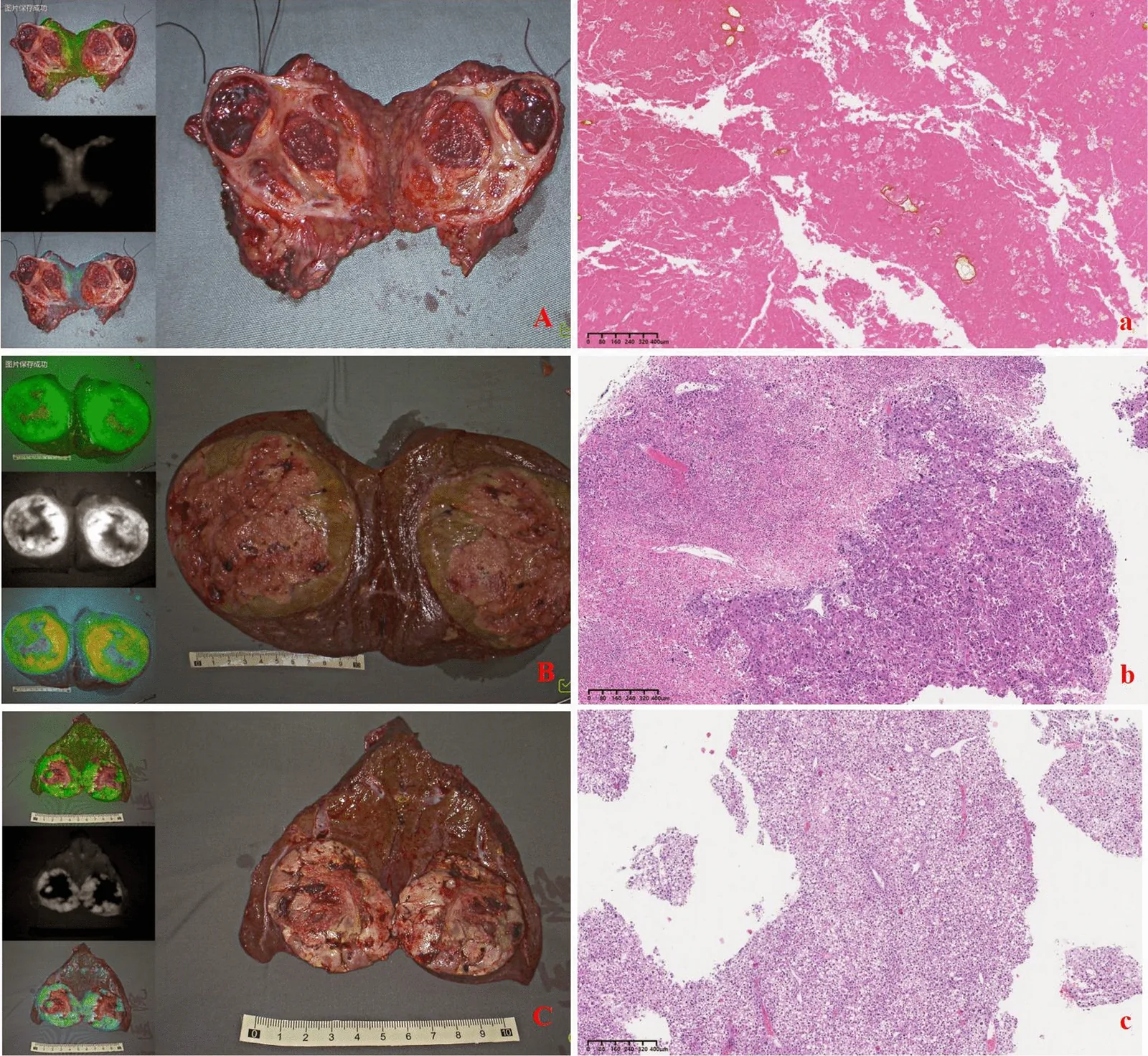
Fluorescence patterns and pathological observation of liver cancer resection specimens. (A(a-400x): Complete necrosis (100%) of the tumor with no fluorescence intensities; B(b-400x), C(c-400x): Partial necrosis (50% and 5%) of the tumor with varying fluorescence intensities)

Each patient underwent at least one sample collection from areas of varying fluorescence intensity within the tumor. A total of 103 samples were obtained from 37 patients, with 34 patients demonstrating discernible fluorescence intensities visible to the naked eye in tumor specimens. These fluorescence intensities were categorized into three levels: strong, weak, and none (Fig. 3). The measured fluorescence intensity ranged from 0 to 278, correlating with necrosis ratios ranging from 0 to 100%. For samples with necrosis rates below 20%, subtypes and degrees of differentiation were observed under a microscope (refer to Table 2). Furthermore, the number of sampling, time and cost required for rapid pathological sampling guided by fluorescence were significantly lower than traditional pathological sampling [(2.81 ± 0.91) vs. (53.84 ± 25.73) pieces; (4.24 ± 0.95) vs. (33.43 ± 13.64) hours; (3954.05 ± 472.93) vs. (11,448.65 ± 3424.47) RMB, all p < 0.05)], benefiting both pathologists and HCC patients.

**Table 2:** Pathological characteristics in 103 samples (Rapid pathological sampling method)

Characteristic	Samples (n = 103)
Fluorescence intensity by instrument	
Strong fluorescence (> 100, ≤ 278)	32(31.07%)
Weak fluorescence (> 0, ≤ 100)	31(30.10%)
No fluorescence (0)	40(38.83%)
Tumor necrosis rate	
≥ 0, < 50%	35(33.98%)
≥ 50, < 100%	25(24.27%)
100%	43(41.75%)
Liver cancer subtypes*(n = 25)	
Microtrabecular	2(8.00%)
Macrotrabecular	6(24.00%)
Compact	10(40.00%)
Pseudoglandular	2(8.00%)
Clear cell	2(8.00%)
Steatohepatitic	1(4.00%)
Macrotrabecular-massive	2(8.00%)
Degree of differentiation*(n = 25)	
Moderately differentiated	17(68.00%)
Poor differentiated	8(32.00%)

^*^For histological observation, only samples with a necrosis rate < 20% are included

Additionally, subgroup analysis was conducted on 3 patients with CPR observed using traditional pathological method. No fluorescence intensity was detected in the tumors and 3 tumor samples were collected from each patient, totaling 9 samples for microscopic observation. Complete tumor necrosis (100%) was observed in all samples, corresponding to a fluorescence intensity of 0, and a sensitivity of 1.

### Predictive value of ICG fluorescence intensity on tumor necrosis ratio

**Fig. 4:**
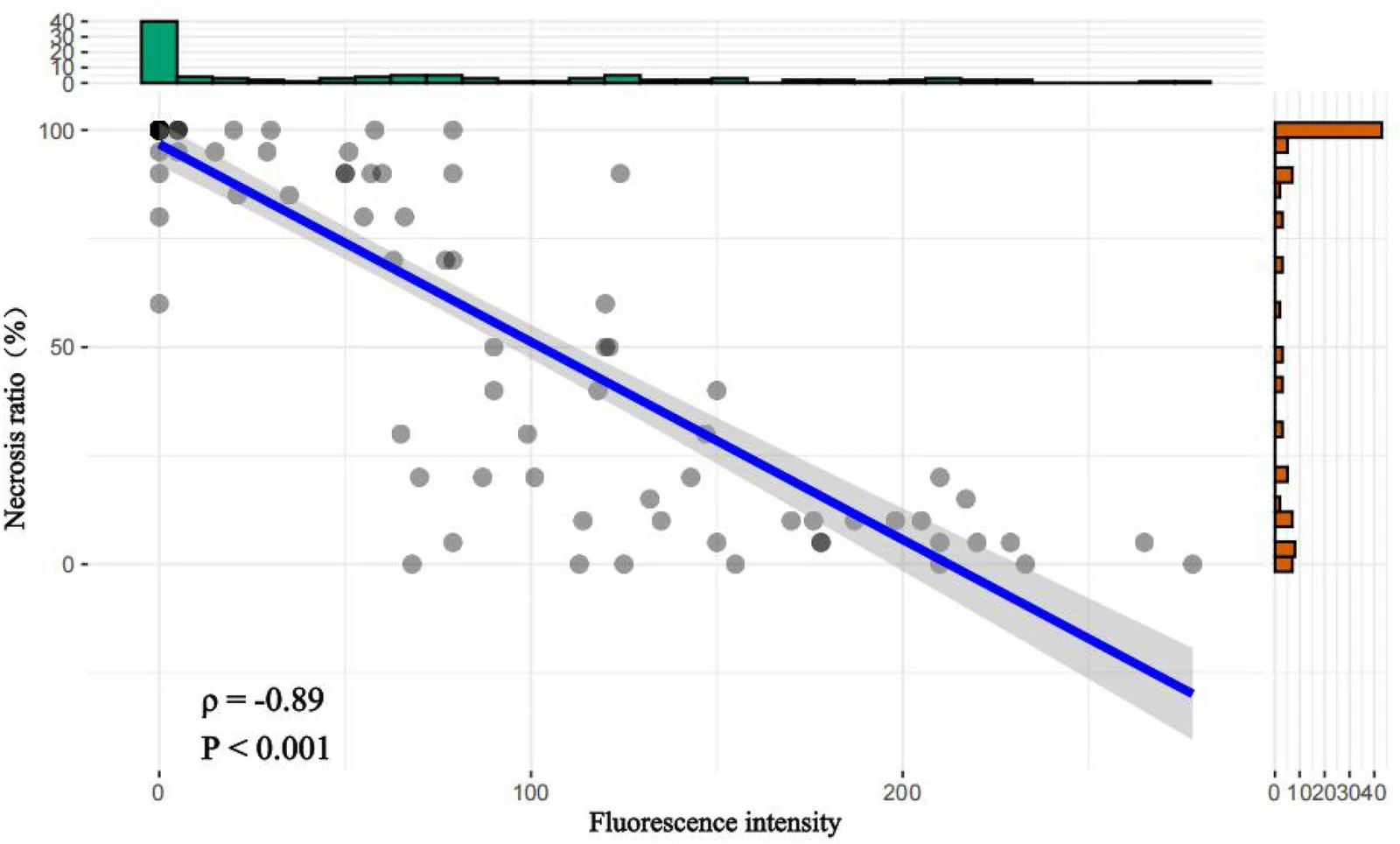
The scatter diagram of the association between ICG fluorescence intensity value and tumor necrosis ratio

The Spearman correlation coefficient of fluorescence intensity value and tumor necrosis ratio was ρ = −0.89 (*p*-value < 0.001), indicating a high association between fluorescence intensity value and tumor necrosis ratio, see Fig. 4.

We used linear regression model to better evaluate the predictive capacity of fluorescence intensity value on tumor necrosis ratio. The results of the linear regression model are summarized on Table 3. The R^2^ of the prediction model is 78.06%, indicating good predictive capacity. The linear regression equation of fluorescence intensity value predicting tumor necrosis ratio is:

**Table 3:** Parameters of linear regression prediction model

	β	S.E	t	P
Intercept	96.784	2.474	39.128	< 0.001
ICG intensity value	−0.456	0.024	−19.074	< 0.001

Tumor necrosis ratio (%) = −0.456*(ICG fluorescence intensity value) + 96.784

To further explore the diagnostic value of ICG fluorescence intensity on tumor necrosis, we employed logistic regression model to test the ability of fluorescence intensity to differentiate complete tumor necrosis and partial tumor necrosis. The results of the logistic regression model showed that fluorescence intensity could significantly predict the tumor necrosis state (complete necrosis or partial necrosis) (β = −0.066, *p*-value < 0.001), see Table 4.

**Table 4:** Parameters of the logistic regression prediction model

	β	SE	Z	P	OR
Intercept	1.868	0.420	4.447	< 0.001	6.4738
ICG intensity	−0.066	0.014	−4.656	< 0.001	0.9363

**Fig. 5:**
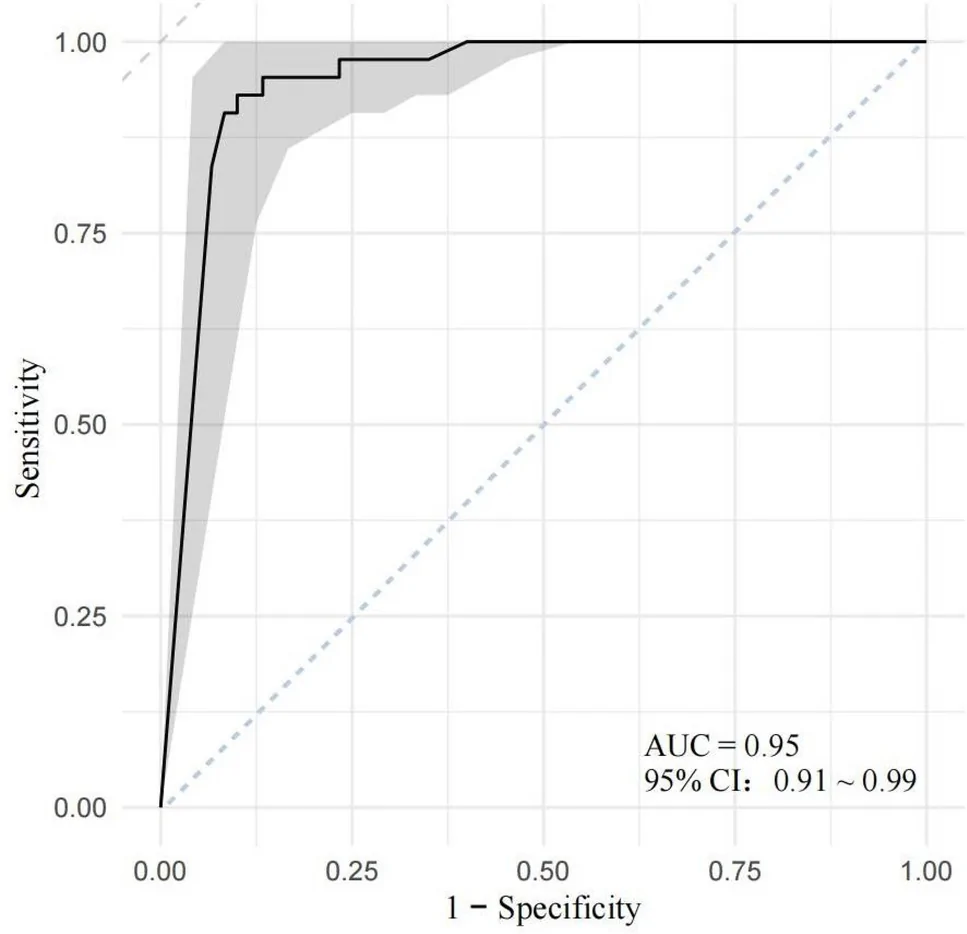
Discrimination of the logistic regression prediction model

In this study, in order to further determine the best discrimination threshold of ICG fluorescence value in predicting complete tumor necrosis, we used the principle of"nearest to the ideal point (0, 1)"based on ROC curve for analysis. Each point in the ROC curve corresponds to a specific discrimination threshold, and its horizontal and vertical coordinates are the false positive rate (1—specificity) and the true positive rate (sensitivity). The theoretical ideal point is (0, 1), that is, the sensitivity (SEN) and specificity (SPE) are both 1. Accordingly, we calculated the Euclidean distance from each point to the ideal point, and selected the threshold corresponding to the minimum distance as the optimal classification point. The results showed that when the ICG fluorescence value was less than 20.5, it was the most effective way to distinguish whether the tumor was completely necrotic or not. The SEN of the threshold was 0.930, and the SPE was 0.900, indicating that the index had a high ability to distinguish (AUC = 0.95, 95% CI 0.91–0.99), see Fig. 5. In addition, we calculated the Youden index, which is 0.830. We also used Liu’s method to identify the threshold maximizing the product of SEN and SPE. With a SEN of 0.930 and SPE of 0.900, the resulting SEN × SPE value of 0.837 confirms the threshold’s reliability in distinguishing necrotic and viable tumor tissue.

**Fig. 6:**
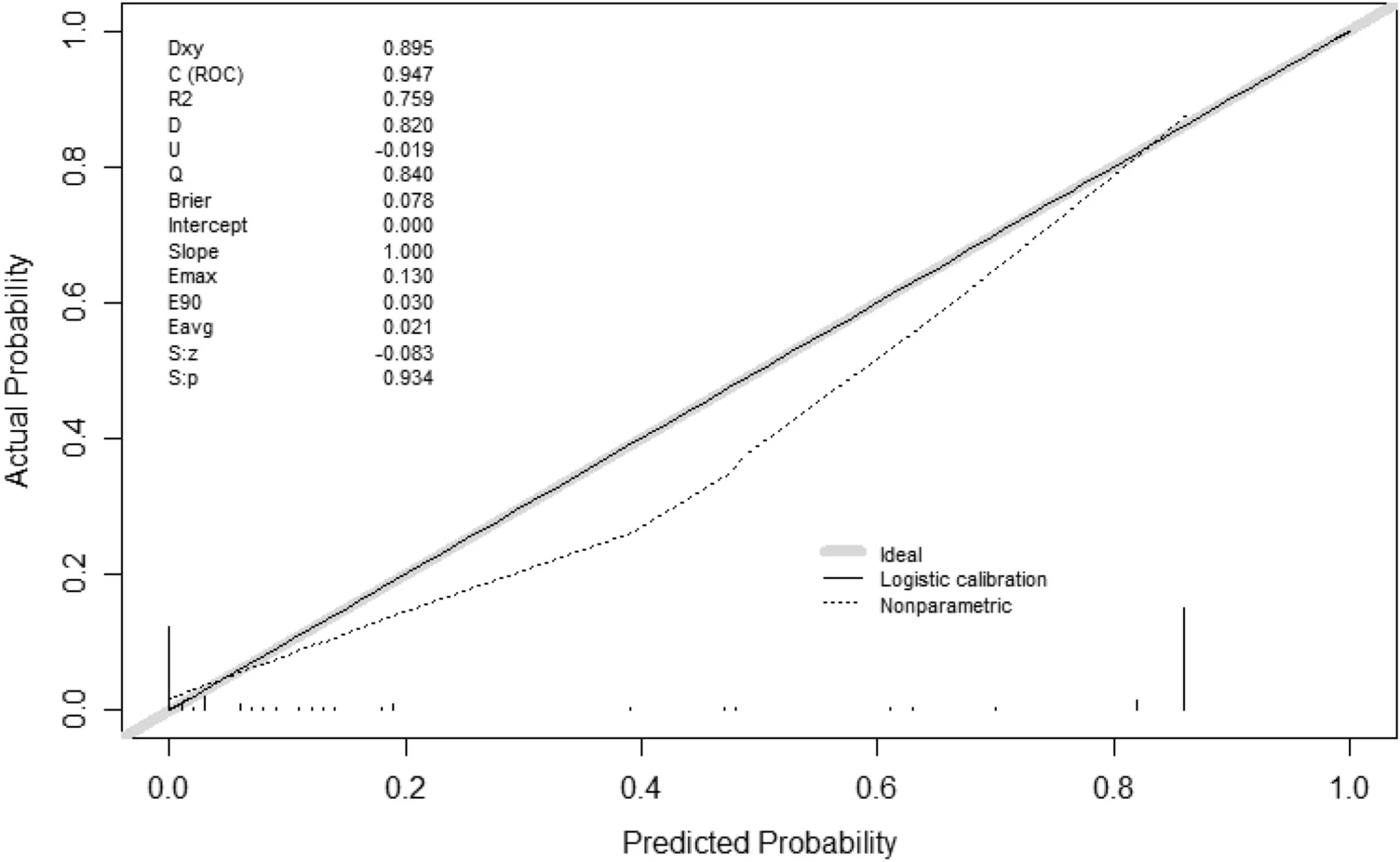
Calibration curve for the logistic regression prediction model

**Fig. 7:**
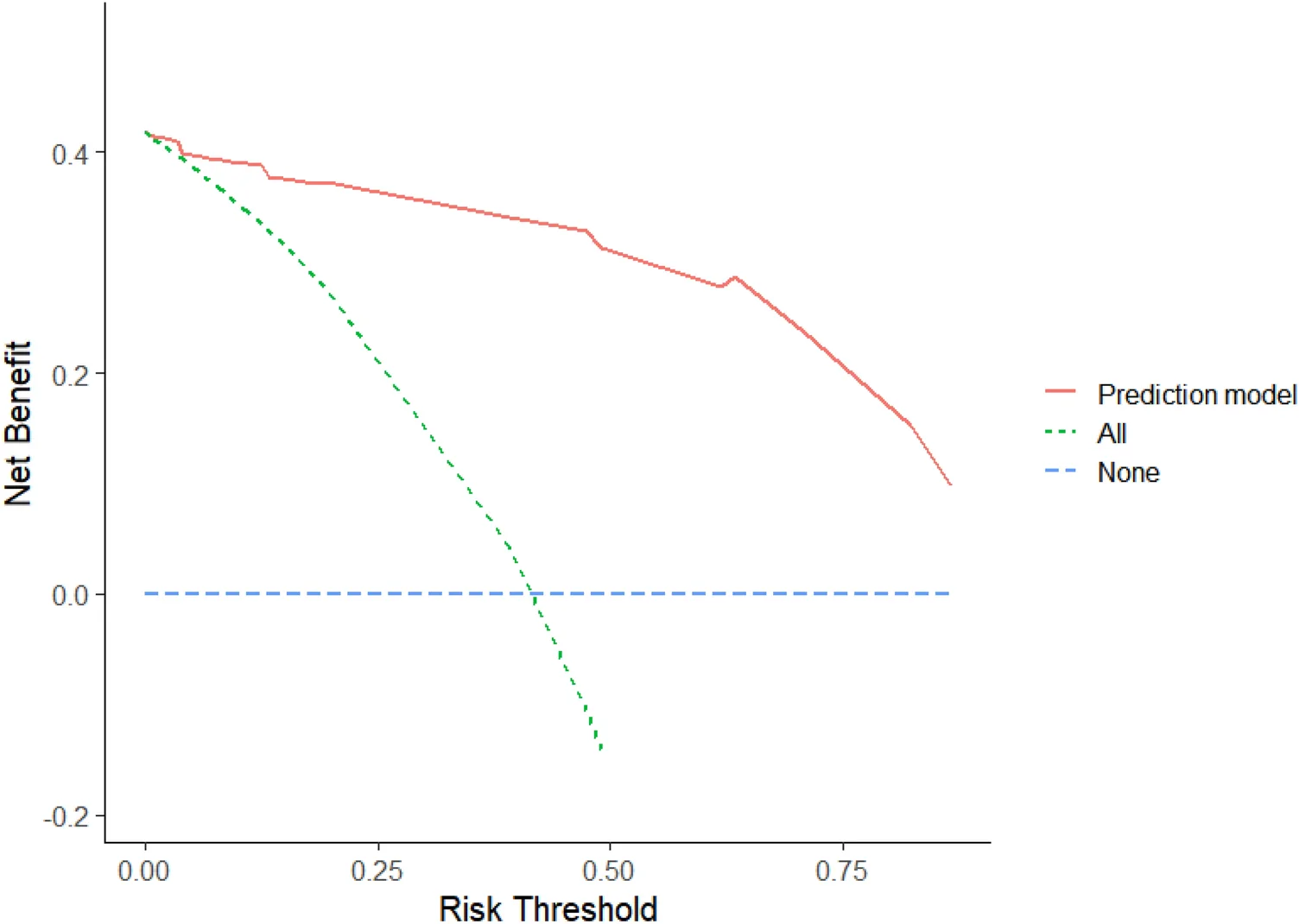
Decision curve analysis for the logistic regression prediction mode

The result of the HL goodness of fit test (χ^2^ = 4.061, df = 8, *p*-value = 0.852) and calibration curve indicated that the prediction model had a good prediction effect requiring no further calibration, see Fig. 6. The DCA curve showed that the prediction model could yield a net benefit to a wide range of approximately between 1 and 87%, indicating this model is beneficial in making decisions in clinical settings, see Fig. 7.

### Long-term prognosis

**Fig. 8:**
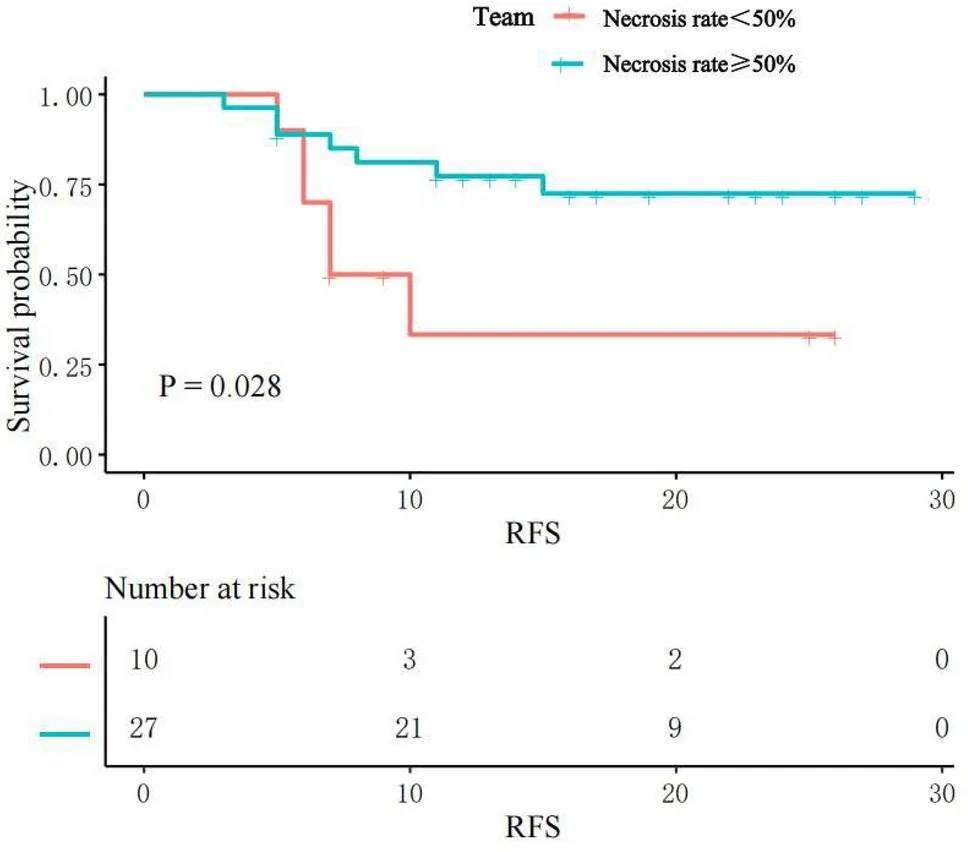
Survival curve analysis of patients with different tumor necrosis rates

Among the enrolled HCC patients, 13 patients had postoperative tumor recurrence, and 2 patients died due to tumor progression. In order to verify the relationship between tumor necrosis rate and prognosis of recurrence free survival (RFS), we grouped the overall necrosis rate of postoperative pathological tumors by 50%, including 27 patients with tumor necrosis rate ≥ 50% and 10 patients with tumor necrosis rate < 50%. Survival analysis by Kaplan–Meier method showed that RFS in patients with tumor necrosis rate ≥ 50% was significantly better than that in patients with tumor necrosis rate < 50% (P = 0.028). The results showed that tumor necrosis rate was closely related to the prognosis of HCC patients, and accurate judgment of tumor treatment response would provide an important reference for postoperative treatment and management of patients, see Fig. 8.

## Discussion

In recent years, the landscape of liver cancer treatment has evolved significantly with the advent of neoadjuvant and conversion therapies, complemented by radiotherapy, immunotherapy, and targeted drug regimens. This paradigm shift has culminated in a holistic treatment approach centered on surgical intervention. Notably, the integration of immune checkpoint inhibitors and molecular targeted drugs, epitomized by the “T + A” regimen [25] in the IMbrave150 trial, has revolutionized the treatment landscape for advanced HCC. Compared to conventional modalities, this combined therapy has substantially extended median survival and progression-free survival while concurrently reducing mortality and disease progression risks by 34% and 35% respectively, heralding an era of precision, amalgamation, and comprehensive care in systemic HCC management.

Liver cancer, characterized by diverse biological behaviors across varying liver disease backgrounds, presents intricate challenges in disease assessment and treatment efficacy evaluation, particularly for pathologists. Preoperative interventions further complicate tumor status assessment, rendering conventional pathology sampling methods inadequate. In response, a novel rapid pathological examination technique utilizing ICG fluorescence imaging for guided sampling was investigated. This cutting-edge technology offers precise tumor localization and rapid differential diagnosis, thereby optimizing sampling accuracy, overcoming diagnostic hurdles, and expediting clinical decision-making compared to traditional approaches.

ICG, which is commonly employed to evaluate liver function reserve, serves as a reliable indicator of the liver’s regenerative capacity following surgery or trauma. By leveraging its distinctive fluorescent properties, the identification of liver cell clearance is enabled, with retention rates serving as surrogate markers for liver function. Given the diverse preoperative treatment modalities, pre-surgical liver function assessment becomes indispensable to ensure surgical feasibility. Furthermore, the impairment of ICG excretion in HCC lesions due to abnormal bile duct development underscores its utility in discerning tumor tissue from normal liver parenchyma [26].

Our findings unveil a significant correlation between fluorescence intensity and tumor necrosis rate, underscoring the method's high sensitivity and specificity in discerning necrotic tissue. Notably, complete pathological remission correlates with the absence of tumor fluorescence, suggesting a potential direct assessment modality for remission status. Additionally, our study elucidated HCC subtypes and differentiation profiles, affirming the universal applicability of ICG across diverse tumor phenotypes. Interestingly, for patients with good preoperative treatment results, such as patients who have achieved major pathological response, there is often a large amount of necrosis within the tumor. In this case, it is particularly important to identify residual tumor cells, which our rapid pathology technology can accurately achieve (Supplement 1).

In addition, ICG can be taken up by hepatocytes across various liver conditions and still produce a fluorescent signal. In our study, surgical specimens from patients with different hepatitis virus infections, liver cancer subtypes, and tumor differentiation grades all exhibited ICG fluorescence. Furthermore, intraoperative bleeding at the tumor cut surface does not interfere with ICG fluorescence imaging. Since ICG is not present in the bloodstream and its fluorescence has a certain tissue penetration capacity, the presence of blood over the tumor section does not obstruct signal detection by the imaging device, thereby ensuring the reliability of the results. ICG fluorescence-guided rapid pathological sampling is suitable for patients undergoing different types of liver surgery.

The patients in our study included those who underwent both anatomical and non-anatomical resections. Extended liver resection may be warranted in cases where tumors invade or are in close proximity to major vascular structures, or when achieving negative surgical margins through standard resection is not feasible. In this context, ICG was primarily used for locating residual tumor tissue and was administered intravenously one week prior to surgery. ICG can also be used for segmental or sectional resection in anatomical hepatectomy, which typically requires a smaller dose administered intraoperatively. These two applications differ in both timing and dosage. When used for rapid pathological sampling, ICG is administered through a peripheral vein, making it unaffected by intrahepatic or perihepatic vascular variations and thus broadly applicable. In contrast, when ICG is used to guide anatomical hepatectomy, it is essential to accurately identify the hepatic vessels supplying the resection area, which may be complicated by anatomical variations in the vasculature [18, 26].

As an observational study, our work does not offer definitive proof of the safety or superiority of ICG-guided rapid pathological evaluation. Instead, it provides preliminary clinical evidence supporting the feasibility and short-term safety of this approach in the intraoperative setting. This study offers several key strengths: it introduces a novel ICG-based technique that enhances intraoperative pathological assessment; improves tissue sampling precision, particularly in challenging diagnostic contexts; and demonstrates broad applicability across diverse tumor profiles and surgical strategies. Nevertheless, prospective, large-scale studies are essential to validate these findings and establish robust, evidence-based recommendations for integrating ICG fluorescence guidance into liver cancer surgery.

## Conclusion

Rapid pathological evaluation guided by ICG fluorescence in liver cancer resection specimens proves as a feasible approach, providing superior tumor localization, improved sampling accuracy, and cost-effective advantages over conventional methods. This groundbreaking innovation holds promise in streamlining clinical treatment pathways and warrants further clinical validation for widespread integration.

## Supplementary Information

Below is the link to the electronic supplementary material.

## Data Availability

All data related to this study are included in this paper. Details are available from the corresponding author on reasonable request.

## Abbreviations

HCC: Hepatocellular carcinoma
ICG: Indocyanine green
ICC: Intrahepatic cholangiocarcinoma
cHCC-CC: Combined hepatocellular-cholangiocarcinoma
HBV: Hepatitis B virus
HCV: Hepatitis C virus
MDT: Multidisciplinary team
TACE: Transarterial chemoembolization
ROC: Receiver operating characteristic
AUC: Area under the curve
HL: Hosmer and lemeshow
DCA: Decision curve analysis
PR: Partial response
CR: Complete response
SD: Stable disease
AFP: α-Fetoprotein
MVI: Microvascular invasion
CPR: Complete pathological response
SEN: Sensitivity
SPE: Specificity
RFS: Recurrence free survival
