# Supplementary material for: Innovative rapid pathological assessment technology for hepatocellular carcinoma following comprehensive treatment guided by indocyanine green fluorescence imaging
Source: Updates Surg. 2025 Jul 7;78(5):2081–92. doi: 10.1007/s13304-025-02301-2 (PMC13538157; doi:10.1007/s13304-025-02301-2)
Supplement: Supplementary file 1 [file 13304_2025_2301_MOESM1_ESM.doc]

Supplement 1. Surgical resection specimen

Residual tumor in fluorescent area and necrotic tissue in non fluorescent area
